# Humic Acids Inhibit Platelet Activation to Reduce Venous Thromboembolism in Mice

**DOI:** 10.1155/2022/6606423

**Published:** 2022-12-21

**Authors:** Hong-Tao Lan, Ya-Ting Zheng, Zhou-Jie Tong, Cong Zhang, Xiao-Yan Cong, Zhi-Hao Wang

**Affiliations:** ^1^The Key Laboratory of Cardiovascular Remodeling and Function Research, Chinese Ministry of Education, Chinese National Health Commission and Chinese Academy of Medical Sciences, The State and Shandong Province Joint Key Laboratory of Translational Cardiovascular Medicine, Department of Cardiology, Qilu Hospital, Cheeloo College of Medicine, Shandong University, Jinan, China; ^2^Department of Geriatric Medicine, Qilu Hospital of Shandong University, Key Laboratory of Cardiovascular Proteomics of Shandong Province, Qilu Hospital of Shandong University, Jinan, China; ^3^School of Nursing and Rehabilitation, Cheeloo College of Medicine, Shandong University, Jinan, China; ^4^Shandong Asia-Pacific Highvarve Organisms Science and Technology Co., Ltd., Jinan, China; ^5^Jinan Kuoda Biotechnology Co., Ltd., Jinan, China

## Abstract

**Objective:**

We aimed to investigate the effects of the natural product humic acids (HA) on platelet activation and development of venous thromboembolism (VTE) in mice and further explore the relevant mechanism.

**Methods:**

Eight-week C57BL/6 mice were randomly assigned to three groups: sham operation group (*n* = 7), VTE group (*n* = 8), and VTE + HA group (*n* = 10). Thrombi were harvested to hematoxylin-eosin staining to evaluate the thrombolysis and recanalization of the thrombus. In addition, flow cytometry was performed to detect the expression levels of protein disulfide isomerase on endothelial-derived exosomes and glycoprotein IIb/IIIa on the surface of the activated platelets surface in plasma. Furthermore, the protein expression level of glycoprotein IIb/IIIa in thrombus was determined by immunohistochemistry and immunofluorescence.

**Results:**

The length of thrombosis in the VTE + HA group was significantly shorter than that in the VTE group (*P* = 0.040). No significant differences were observed in thrombolysis and recanalization between the VTE + HA group and the VTE group (*P* > 0.05). The content of protein disulfide isomerase carried by endothelial-derived exosomes was significantly increased in the VTE group (*P* = 0.008) but significantly reduced by native humic acids (*P* = 0.012). Compared with the VTE group, the expression of glycoprotein IIb/IIIa on activated platelet surface in the VTE + HA group was significantly decreased (*P* = 0.002). The concentration of plasmatic P-selectin in the VTE group was significantly higher than that in the VTE + HA group (*P* < 0.001).

**Conclusion:**

We demonstrate that HA possess a pharmacological property that decreases venous thrombus formation in mice. The underlying mechanism is that HA could inhibit the expression of glycoprotein IIb/IIIa on the activated platelets surface by suppressing endothelial-derived exosomes carrying on protein disulfide isomerase, thereby blocking platelet activation.

## 1. Introduction

Venous thromboembolism (VTE) is a potential pathogenic disease that results in venous obstructive reflux disorder and a series of related pathophysiological changes owing to intravenous thrombosis, comprising deep vein thrombosis (DVT) and pulmonary embolism (PE). Approximately, 10 million cases occur every year around the world [1]. Venous thromboembolism, defined as the third most frequent acute cardiovascular event, has been a considerable contributor to the burden of noncommunicable diseases [2]. The annual incidence of VTE is estimated to be 0.1%–0.2%, which will continue to rise exponentially with the longer life expectancy of the population worldwide [3, 4].

As a chronic recurrent illness, VTE is concerned with fatality, hemorrhage related to anticoagulant therapy, and perennial disability. Except for recurrent thrombosis and anticoagulation-related bleeding, both postthrombotic syndrome and chronic thromboembolic pulmonary hypertension are increasingly recognized as serious complications of VTE, which impose significant morbidity and reduce the quality of life [5, 6]. The numerous guidelines unanimously recommend that anticoagulation is the basic therapeutic schedule for VTE. Direct oral anticoagulant is the frontline treatment for almost all individuals with VTE. Given the advantages of rapid onset and definitive effectiveness in the prevention and treatment of thrombosis, the new oral anticoagulant was recommended to treat venous thromboembolism in the 10th edition of the “Guidelines for Antithrombotic Therapy of Venous Thromboembolism” published by the American College of Chest Physicians in 2016. Nevertheless, the risk of bleeding and recurrence of VTE remains an unresolved issue. Patients presenting with VTE for the first time who completed minimum 3 months of anticoagulation had a 10% risk of recurrent VTE in the first year after treatment, 16% in two years, 25% in five years, and 36% in ten years [7]. In this situation, antiplatelet therapy will become another critical approach.

Classical theory believes that platelets are mainly involved in the formation of the arterial thrombus. Although platelets are also contained in venous thrombosis, fibrin and erythrocytes dominate. In recent years, the role of platelets in VTE has been gradually emphasized, attributing to the emergence of increasing studies on platelet complex signal transduction, intercellular interaction, and related proteins in platelets [8–10], as well as the clinical cognitive transformation of antiplatelet drug aspirin to prevent VTE [11].

The pathogenesis of arteriovenous thrombosis is intertwined to a large extent. It has been proposed that individuals diagnosed with VTE are at an elevated risk of subsequent arterial ischemic incidents [12, 13]. In the same way, atherosclerotic disease predisposes individuals to venous thrombosis [14]. Some studies in animal models have verified the key role of platelet activation in the pathogenesis of VTE [15, 16]. Additionally, the clinical evidence from observational studies also supports the importance of platelet reactivity in venous thrombosis [17]. In addition, antiplatelet drugs have been employed in preventing and treating VTE in recent years [18]. The joint application of antiplatelet and anticoagulation has a superimposed enhancement effect, which is obviously beneficial to VTE treatment, especially for some VTE patients accompanied by an arterial vascular disease. Yet, the combination brings about an amplified incidence of bleeding complications.

In view of the shortcomings of current drugs used to prevent thrombotic diseases such as increased bleeding risk and single target of action leading to tolerance, it is imperative to develop a new type of antithrombotic drug with fewer side effects.

In recent decades, much attention in medical research has been paid to the medicinal properties of natural products, such as humic substances. Some of its pharmacological properties and potential mechanisms of action have been put forward and demonstrated by experiments [19, 20]. The range of diseases treated with humic substances has widely expanded, especially in the areas of major diseases and multiple diseases, encompassing acquired immunodeficiency syndrome [21] and colorectal cancer [22]. Humic acid is a species of the humus substance formed by the natural degradation of animal and plant remains through a series of complex geochemical reactions. It is an organic acid that contains high amounts of aromatic carbon, phenols, and carbohydrate residues. Studies have found that organic acids and polyphenols are natural antithrombotic factors with low toxicity and less side effects, which can significantly lower the danger of thrombosis [23].

Native humic acid is a nontoxic, odorless, noncorrosive, and water-soluble multifunctional polymer compound chemically extracted from lignite, weathered coal, and peat. Classified as a kind of Chinese herbal medicine with the functions of antivirus [24], antitumor [25], anti-inflammatory [26], hemostasis, and wound healing [27], HA has been applied as a drug for multiple diseases in many countries over thousands of years.

So far, no published reports have been found on whether native humic acids can be used for antithrombotic therapy and studies about the influence of HA on platelet activation are lacking. Therefore, we presently investigated the efficacy of native humic acids in venous thrombosis formation and the underlying mechanism in mice.

## 2. Materials and Methods

### 2.1. Preparation of Purified Humic Acids

The lignite samples were originated from Shandong Asia-Pacific Highvarve Organisms Science and Technology Co., Ltd. The preparation process of purified humic acids was performed according to the methods of references [28, 29]. In brief, the lignite samples were dried at 65°C for 2 hours and then washed with distilled water thrice and filtered. The filtrate residue was treated with 25% nitric acid (solid-liquid ratio l : 5) for 40 minutes and then filtered again. The filtrate residue was taken and dissolved in a mixture solution of 2.25% NaOH: Na_2_CO_3_ = 1 : 1 at 55°C for 60 minutes (solid-liquid ratio 1 : 10), stirring once every 10 minutes. Centrifugation was performed at 4000 rpm for 25 minutes. In order to precipitate a brown-black sample residue, strong acid was added to the supernatant collected to adjust PH = 2. After filtration, the sample residue was washed with distilled water 3–5 times. Finally, the precipitate was dried to a constant weight at 65°C and then crushed, sealed, and stored for future use.

### 2.2. Venous Thromboembolism Mice Model

Eight-week-old C57BL/6 mice were purchased from SPF Biotechnology Co., Ltd (Beijing). All mice were raised under controlled conditions (22°C, 12/12 h light-dark cycle) and fed with adequate water and food. Before receiving treatment, animals were randomly distributed into three groups after at least three days of adjustable feeding: the sham operation group (*n* = 7), the VTE group (*n* = 8), and the VTE + HA group (*n* = 10). Based on our preliminary experiment, the mortality rates in the sham operation, VTE, and VTE + HA groups were 16.7%, 28.6%, and 40% respectively; accordingly, 7, 8, and 10 mice were recruited in each group. The sham operation group and VTE group were not given any drug treatment. Native humic acids were added to the drinking water of the VTE + HA group for 40 days (600 mg extract was dissolved in 1 L drinking water and vibrated until it was completely dissolved). A VTE model was established after the administration of the treatment.

Mice were anesthetized by intraperitoneal injection of sodium pentobarbital (1%, 50 mg/kg) and fixed in the supine position. Then, we began preoperative skin sterilization of the surgical area. Subsequently, the abdomen was opened along the linea alba. The inferior vena cava (IVC) was slightly detached from the aorta and then ligated with a 7-0 polypropylene suture over a spacer (30-gauge needle) under the left renal vein. Afterward, the spacer was debrided to shape 90% of the stenosis without disrupting the endothelium. All the lateral branches visible were fully ligated. At last, the bowel was placed back into the cavum abdominis and the peritoneum and skin were stitched with 6-0 sutures. 48 h after the ligation, we reopened the abdominal cavity of the mice, clamped the blood vessel 2 cm below the ligation line, and the thrombus was removed after opening the lumen. A similar procedure, but no IVC ligation was performed on mice in the sham operation group.

### 2.3. Histology, Immunohistochemical Staining, and Immunofluorescence

The methodology here refers to the published literature [30]. Tissues including the thrombus and surrounding vein walls were removed after sacrifice and fixed in 4% formaldehyde, dehydrated through a concentration gradient of ethanol, transparent in xylene, and embedded in paraffin. Thin tissue sections (5 *μ*m thick) harvested on day 2 post-modeling underwent stain with hematoxylin-eosin (HE) and were observed under a light microscope. For immunohistochemical staining, after antigen retrieval by microwave heating in citrate buffer (Beyotime, P0081), sections were blocked with 5% bovine serum albumin (BioFroxx, 4240GR005) before anti-CD41 (Abcam, ab134131) incubation at 4°C overnight. Subsequently, the goat anti-rat IgG secondary antibody (ZGBBT, ZB-2301) was applied for 1 hour at room temperature (RT) followed by the use of diaminobenzidine substrate to visualize the stain sections. The presence of brown spots in the image showed positive staining. Antibodies against CD62P (Proteintech, 60332) and CD41 were employed for immunofluorescence double-staining. The slides were incubated with Dylight 488-conjugated goat anti-rat IgG secondary antibodies (Abbkine, A23220) or Dylight 594-conjugated goat anti-mouse IgG secondary antibodies (Abbkine, A23410). 5 to 8 randomly unrepeated field areas were selected to view under ×400 magnification in an optical microscope.

### 2.4. Isolation of Mice Platelets

On the basis of reference [31], whole blood from mice was pooled in anticoagulation tubes containing sodium citrate. Platelet-rich plasma (PRP) was obtained through centrifugation at 800 rpm for 10 minutes at RT, and platelets were separated from PRP by centrifugation at 2300 rpm for 10 minutes. The supernatant was collected as platelet-rich plasma for subsequent use. Then, platelets were resuspended in an appropriate volume of Tyrode's buffer, adjusting the platelet concentration to 5 × 10^8^ mL.

### 2.5. Flow Cytometry

The content of protein disulfide isomerase (PDI) on the surface of endothelial-derived exosomes (EMP) was determined through APC-CD144 and FITC-PDI dual-color flow cytometry (FCM). APC-CD144 is labeled as exosomes derived from endothelial cells, and EMP^+^PDI^+^ represents PDI carried by exosomes derived from endothelial cells. Briefly, 10 *μ*L plasma was put into a BD tube, along with a 3 *μ*L anti-mouse APC-CD144 antibody (eBioscience, 17-1441-82) and 3 *μ*L anti-mouse FITC-PDI antibody (Santa Cruzm, 74551); then, 84 *μ*L phosphate-buffered salines (PBS) was added to obtain a total volume of 100 *μ*L. Antibodies and samples were incubated for 30 minutes at RT and protected from light. Lastly, 200 *μ*L 4% paraformaldehyde was added. The samples were blended and assayed directly by FCM analysis (BD, FACSAriall, USA).

To assay the expression level of GPIIb/IIIa on the platelet surface, 20 *μ*L platelet suspension (5 × 10^8^ mL) along with 3 *μ*L FITC-CD41 (Biolegend, 133903), 3 *μ*L PE-CD62p (Biolegend, 148306), and 2 *μ*L APC-CD61 (Biolegend, 104316) was placed in a 100 *μ*L system based on PBS to a BD tube. Then, the sample and antibodies mixture was incubated at RT for 30 minutes in the dark. Finally, 200 *μ*L 4% paraformaldehyde was added to fix the platelets before mixing, and the samples were directly tested by FCM analysis (BD, FACS Caliber, USA).

### 2.6. Enzyme Linked Immunosorbent Assay (ELISA)

Mice apical blood was gathered in anticoagulant tubes and centrifuged at 3000 rpm at 4°C for 15 minutes to obtain plasma. The levels of soluble P-selectin in the plasma of each group of mice were determined by a Mouse P-Selectin ELISA Kit (Abcam, Ab200014). The quantitative detection of the von Willebrand factor (vWF) in samples of plasma was done by a Mouse vWF ELISA Kit (LifeSpan Bioscience, LS-F22891). The whole operation was strictly implemented following the procedures in the kit instructions.

### 2.7. Statistical Analysis

All experimental data were expressed as means ± SEM. Statistical data were processed using independent samples *t*-test or one-way ANOVA in SPSS version 26 software and statistical graphs were presented using GraphPad Prism 6.0. Statistical significance was considered when *P* < 0.05.

## 3. Results

### 3.1. Length of Thrombi

**Figure 1:**
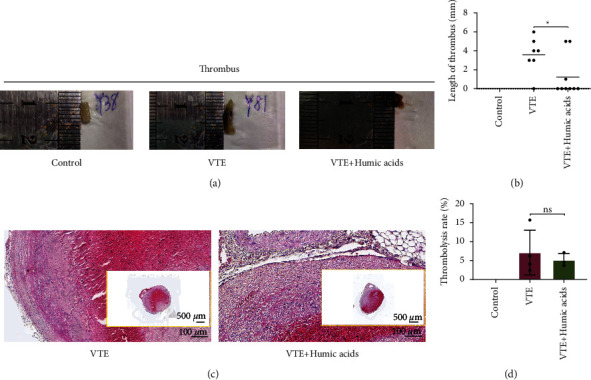
Length and dissolution of thrombus. (a) Picture of the thrombus. (b) Length of the thrombus. (c) Picture of thrombus dissolution. (d) Thrombolysis rate. Statistical analyses were performed using independent samples *t*-test. Date represent mean ± SEM. ^*∗*^*P* < 0.05. HE, hematoxylin eosin staining. NS, no significance.

The survival status of mice and inferior vena cava thrombosis 2 days after modeling are shown in Table 1. Thrombosis was found in the VTE group and VTE + HA group, but no thrombus was found in the sham operation group, proving the flow restriction model surgery method can be successfully modeled (Figure 1(a)). Moreover, the thrombi length of the VTE + HA group was significantly shorter than that of the VTE group (*P* = 0.040, Table 2, Figure 1(b)).

### 3.2. Thrombolysis and Recanalization

Forty days after treatment with humic acids, the thrombus was harvested to HE staining to assess the thrombolysis and recanalization of the thrombus. Since no thrombosis was in the sham operation group, we only compared the HE staining results of the VTE group and VTE + HA group. There was not statistically significant evidence to show the difference between the two groups in terms of the distance from the thrombus to the venous wall (*P* > 0.05, Figures 1(c) and 1(d)).

### 3.3. Expression of PDI on Endothelial-Derived Exosomes in VTE Is Diminished by Humic Acids

**Figure 2:**
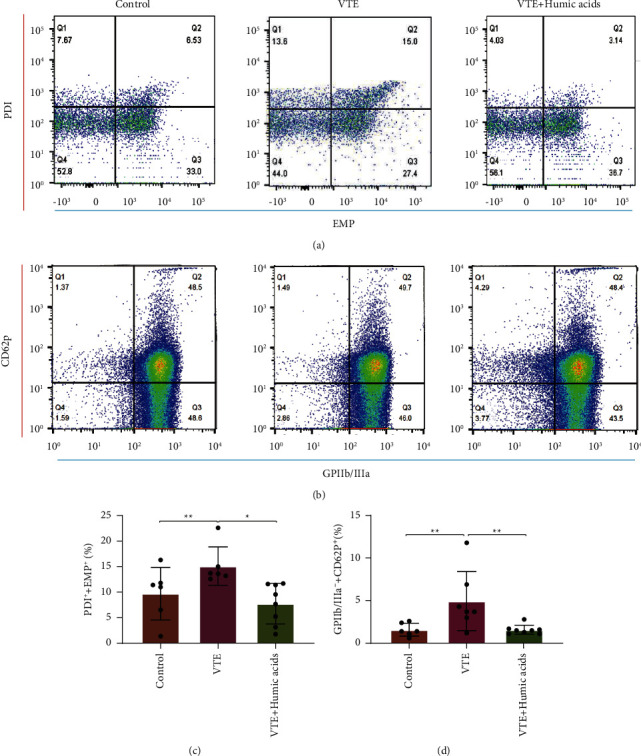
PDI expression on endothelium-derived exosome and GPIIb/IIIa expression on activated platelets. (a) FCM assay of PDI-FITC expression on endothelial-derived exosome marked by CD144-APC. (b) FCM assay of platelet marked by CD41-FITC and CD62p-PE in mice. (c) and (d) FCM assay. Statistical analyses were performed using one-way ANOVA. Date represent mean ± SEM. ^*∗*^*P* < 0.05, ^*∗∗*^*P* < 0.01.

The presence of PDI on the surface of endothelial-derived exosomes was detected through a dichromatic FCM of CD144-APC and PDI-FITC. The level of PDI positive and CD144 positive exosomes was significantly enhanced in the VTE group in contrast to the sham operation group (*P* = 0.008, Figures 2(a) and 2(c)), and a significant decrease was observed in PDI carried by exosomes derived from endothelial cells in the VTE + HA group than the VTE group (*P* = 0.012, Figures 2(a) and 2(c)). While PDI content on the surface of the endothelial-derived exosome in plasma was reduced in the VTE + HA group compared with the sham operation group, the difference is not statistically significant (*P* > 0.05, Figures 2(a) and 2(c)).

### 3.4. Expression of GPIIb/IIIa on the Surface of Activated Platelets Is Downregulated by Humic Acids

Expression of GPIIb/IIIa on the activated platelet surface in plasma was tested by dichromatic FCM of CD41-FITC and CD62-PE. CD62P is a marker of platelet activation. We found that the expression of activated platelet surface GPIIb/IIIa in plasma in the VTE + HA group was markedly decreased compared with the VTE group (*P*=0.006, Figures 2(b) and 2(d)), whereas the expression level was significantly higher in the VTE group than in the sham operation group (*P*=0.009, Figures 2(b) and 2(d)). Also, there was no significant difference in the expression of GPIIb/IIIa on the surface of activated platelets in plasma between the VTE + HA group and the sham operation group (*P* > 0.05, Figures 2(b) and 2(d)).

**Figure 3:**
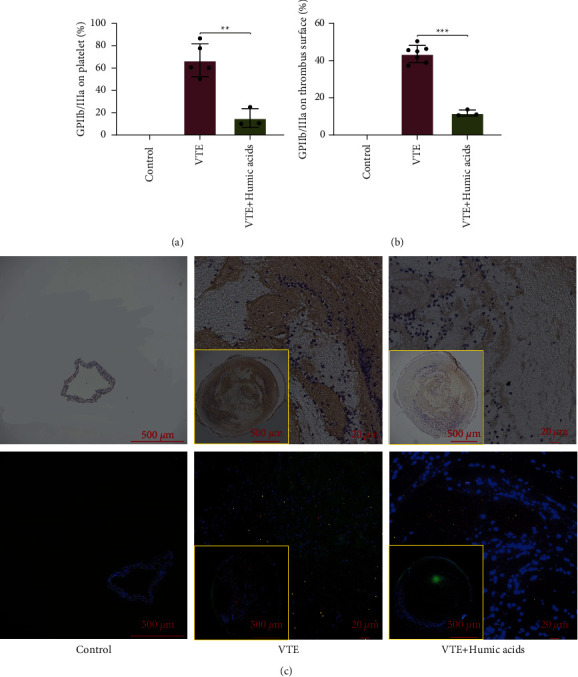
GPIIb/IIIa expression in thrombus. (a) and (b) GPIIb/IIIa expression assay. (c) Expression of GPIIb/IIIa observed by IHC staining. Expression of GPIIb/IIIa observed by IF staining. Statistical analyses were performed using independent-samples*t*-test. Date represent mean ± SEM. ^*∗∗*^*P* < 0.01, ^*∗∗∗*^*P* < 0.001. IHC, immunohistochemistry; IF, immunofluorescence.

Besides, the thrombus removed from the veins of the mice in the VTE group and VTE + HA group was subjected to immunohistochemistry and immunofluorescence, which were applied to measure the level of total GPIIb/IIIa in thrombus and the expression of GPIIb/IIIa on the activated platelet surface in thrombus, respectively. Both histopathological tests showed the same trend, that is, the expression level of GPIIb/IIIa whether in the thrombus or on the surface of activated platelets in the thrombus was significantly lower in the VTE + HA group compared with the VTE group (*P*_IHC_ < 0.001, *P*_IF_ = 0.002, Figures 3(a)–3(c)).

### 3.5. Plasma Coagulation Factor Is Influenced by Humic Acids

**Figure 4:**
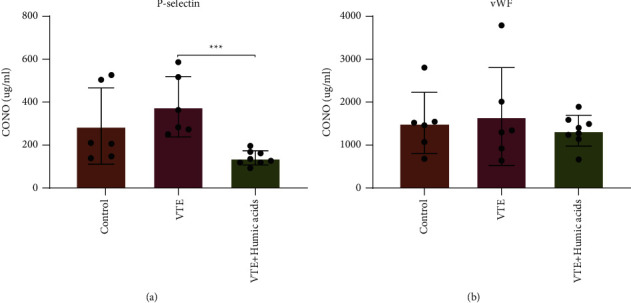
Platelet activation index expression in plasma. (a) Plasma P-selection level in mice detected by ELISA. (b) Plasma vWF level in mice detected by ELISA. Statistical analyses were performed using one-way ANOVA. Date represent mean ± SEM. ^*∗∗∗*^*P* < 0.001. vWF, von Willebrand factor.

Plasma P-selectin and vWF concentrations were measured by ELISA in order to ascertain the influence of native humic acids on coagulation. P-selectin of the VTE + HA group was significantly decreased in comparison with the VTE group (*P* < 0.001, Figure 4(a)). However, the concentration of plasmatic P-selectin in the VTE group was not significantly higher than in the sham operation group (*P* > 0.05, Figure 4(a)). No significant difference was discovered in plasmatic vWF concentrations among the three groups (*P* > 0.05, Figure 4(b)).

## 4. Discussion

Herein, our findings demonstrate the therapeutic potential of native humic acids on VTE. Admittedly, the definitive shared segment of platelet activation is GPIIb/IIIa. When GPIIb/IIIa is activated, then it can bind to fibrinogen and connect to adjacent platelets through fibrinogen, causing platelets to aggregate and form early thrombosis. The significantly increased expression of GPIIb/IIIa on activated platelets of the VTE group illustrates that platelets are activated in the VTE state and perform a pivotal function in venous thrombosis. Furthermore, it is proven that native humic acids take an inhibitory effect on thrombus formation rather than on thrombus recanalization, and such an effect is achieved in part by inhibiting the platelet activation-dependent EMP-PDI and GPIIb/IIIa signaling pathway. The concentration of P-selectin in plasma was significantly decreased by HA, further confirming that native humic acids have the function of restraining platelet activation.

The classical perspective put forward by Virchow believes that variations in blood flow velocity, changes in blood composition, and vessel wall damage are the three primary elements that trigger VTE. Damage and activation of endothelial cells promote the formation of thrombus and significantly affect the initial link of thrombus generation. Injury to the venous intima activates the release of thrombin, ADP, and other procoagulant substances and at the same time promotes the release of microparticles through tissue factors outside the blood vessel. P-selectin glycoprotein ligand 1 induces the binding of P-selectin to its specific receptors [32, 33]. On the other hand, the microparticles transport tissue factor (TF) to bind with P-selectin, colonize the surface of platelets and endothelial cells, activate platelet aggregation, and promote local fibrous deposition and the formation and development of thrombus.

GPIIb/IIIa acts as the final pathway for platelet aggregation and has an influential role in thrombosis. Previous studies have confirmed that exosomes derived from platelets and endothelial cells carry PDI [34, 35]. PDI on the platelet surface can regulate the isomerization of the GPIIb/IIIa receptor disulfide bonds and promote changes in the spatial structure of GPIIb/IIIa receptors, thereby activating the GPIIb/IIIa receptor, which activates platelets and facilitates thrombosis [36]. When vascular endothelial cells are damaged, endothelial-derived exosome is released, which carries PDI. EMP-PDI activates platelets by binding to GPIIb/IIIa receptors on the surface of platelets, releasing more platelet-derived exosomes carrying PDI and realizing the platelet cascade amplification signal conduction.

Based on the role of PDI in regulating thrombosis, people regard it as a new anti-thrombotic target. However, PDI inhibitors are still in clinical trials and the therapeutic risks associated with bleeding remain to be established. The antiplatelet activity of numerous Chinese herbal medicines and their multiple mechanisms of action have recently been highlighted. As a traditional Chinese medicine, native humic acids have attracted our attention in this study because it contains polyphenols, a natural antithrombotic factor. An in vitro experiment has shown that polyphenols can modulate platelet function and suppress many pathways related to platelet activation and aggregation [37]. Similarly, our study also revealed that native humic acids inhibited platelet activation to reduce venous thrombus formation, and its potential mechanism was to decrease the expression of GPIIb/IIIa on the activated platelet surface by inhibiting EMP-PDI.

### 4.1. Study Limitations

Even though it can be deemed that our study results so far are promising, the clinical safety and tolerability of HA remain to be investigated. Furthermore, studies are warranted to explore the effect of PDI carried by exosomes derived from endothelial cells on the structural alteration of GPIIb/IIIa.

## 5. Conclusions

In spite of not a few studies have been conducted on HA, a polyphenolic compound, there are no data found in published reports mentioning its effect on thrombosis. On the basis of this study, compared to the group with no added humic acids, a remarkable improvement was manifested in the group that it was added to. Our results suggest that dietary supplementation of HA is a considerable mode to inhibit platelet activation and antithrombosis. The underlying mechanism is concerned with inhibiting the expression of GPIIb/IIIa on the activated platelet surface by a restraint of PDI on endothelial-derived exosomes. It provides a novel antithrombotic method that may be used for VTE treatment.

## Figures and Tables

**Table 1:** Survival condition of mice 2 days after operation.

Group name	Total	Death	VTE	VTE formation ratio (%)
Control	7	0	0	0
VTE	8	1	6	85.7
VTE + HA	10	1	3	33.3

**Table 2:** The length of thrombi in each group.

Group name	Length (cm)
Control	0
VTE	0.36 ± 0.190
VTE + HA	0.12 ± 0.217^*∗*^

^*∗*^=*P* < 0.05 vs. VTE group.

## Data Availability

The data supporting the results of this study can be obtained from the corresponding authors upon reasonable request.

## Supplementary Materials

Supplementary Materials Table S1: the key resources used in this experiment.
