# Supplementary material for: Humic Acids Inhibit Platelet Activation to Reduce Venous Thromboembolism in Mice
Source: Evid Based Complement Alternat Med. 2022 Dec 21;2022:6606423. doi: 10.1155/2022/6606423 (PMC9797308; doi:10.1155/2022/6606423)
Supplement: Supplementary Materials — Table S1: the key resources used in this experiment. [file 6606423.f1.docx]

**Table S1: The key resources used in this experiment.**

| REAGENT or RESOUCE | SOURCE | IDENTIFIER |
| --- | --- | --- |
| Antibodies |  |  |
| Anti-CD41 | Abcam | Ab134131 |
| Anti-CD62P | Proteintech | 60322 |
| Goat anti-rat IgG secondary antibody | ZGBBT | ZB-2301 |
| Dylight 488, Goat Anti-Rabbit IgG | Abbkine | A23220 |
| Dylight 594, Goat Anti-Mouse IgG | Abbkine | A23410 |
| anti-mouse APC-CD144 antibody | eBioscience | 17-1441-82 |
| anti-mouse FITC-PDI antibody | Santa Cruzm | 74551 |
| anti-mouse FITC-CD41 antibody | Biolegend | 133903 |
| anti-mouse PE-CD62p antibody | Biolegend | 148306 |
| anti-mouse APC-CD61 antibody | Biolegend | 104316 |
| Critical Commercial Assays |  |  |
| Mouse P-Selectin ELISA Kit | Abcam | Ab200014 |
| Mouse vWF ELISA Kit | LifeSpan Bioscience | LS-F22891 |
| Chemicals |  |  |
| bovine serum albumin | BioFroxx | 4240GR005 |
| citrate antigen repair solution | Beyotime | P0081 |
| DAB chromogenic kit | Servicebio | G1212 |
| NaOH | Sigma-Aldrich | S5881 |
| Na_2_CO_3_ | Sigma-Aldrich | S6014 |
